# Number of visits to the emergency department and risk of suicide: a population- based case–control study

**DOI:** 10.1186/s12889-015-1544-5

**Published:** 2015-03-07

**Authors:** Runar Bragi Kvaran, Oddny Sigurborg Gunnarsdottir, Adalbjorg Kristbjornsdottir, Unnur A Valdimarsdottir, Vilhjalmur Rafnsson

**Affiliations:** Landspitali the National University Hospital, Reykjavik, Iceland; Office of Education, Research and Development, Landspitali the National University Hospital, Reykjavik, Iceland; The Centre of Public Health Sciences, University of Iceland, Reykjavik, Iceland; Department of Preventive Medicine, University of Iceland, Reykjavik, IS-101 Iceland

**Keywords:** Suicide, Number of visits, Population-based, Case–control study, Risk factors, Discharge diagnosis

## Abstract

**Background:**

The aim was to study whether number of visits to emergency department (ED) is associated with suicide, taking into consideration known risk factors.

**Methods:**

This is a population-based case–control study nested in a cohort. Computerized database on attendees to ED (during 2002–2008) was record linked to nation-wide death registry to identify 152 cases, and randomly selected 1520 controls. The study was confined to patients attending the ED, who were subsequently discharged, and not admitted to hospital ward. Odds ratio (OR) and 95% confidence intervals (CI) of suicide risk according to number of visits (logistic regression) adjusted for age, gender, mental and behavioral disorders, non-causative diagnosis, and drug poisonings.

**Results:**

Suicide cases had on average attended the ED four times, while controls attended twice. The OR for attendance due to mental and behavioral disorders was 3.08 (95% CI 1.61-5.88), 1.60 (95% CI 1.06-2.43) for non-causative diagnosis, and 5.08 (95% CI 1.69-15.25) for poisoning. The ORs increased gradually with increasing number of visits. Adjusted for age, gender, and the above mentioned diagnoses, the OR for three attendances was 2.17, for five attendances 2.60, for seven attendances 5.97, and for nine attendances 12.18 compared with those who had one visit.

**Conclusions:**

Number of visits to the ED is an independent risk factor for suicide adjusted for other known and important risk factors. The prevalence of four or more visits was 40% among cases compared with 10% among controls. This new risk factor may open new venues for suicide prevention.

## Background

The healthcare system can play an important role in the prevention of suicide [1] at least for prospective suicide victims that have had contact with healthcare services prior to their death [2,3]. These healthcare contacts prior to suicide have been reported in a few descriptive studies with respect to the time when they occur prior to the suicide, with the main focus on hospital admission, mental health services, primary healthcare, and general practitioners. Analytical studies on this issue are rare. Three case–control studies on the use of health services before death by suicide [4-6] have reported varying results. The first, based on information from clinics of an American Indian reservation, Midwest United States, found that suicide cases were less likely to be in contact with clinical services than controls [4]; the second, nested in general practitioners practices in the United Kingdom, found that the number of attendances immediately before death did not differ from the control subjects [5]; and the third study, nested in the general population of Alberta, Canada, found that suicide victims had more than twice the number of healthcare visits than controls [6].

Fortunately suicide is a rare event, and on average a general practitioner might have one patient die by suicide in three or four years [7]. Meanwhile, attendees to the emergency departments (ED) have been reported to be at a considerably increased risk of suicide compared with those not attending [6]. Thus the EDs can be viewed as a venue for suicide prevention [8,9]. Screening for suicide in the ED has been suggested and investigated [8,10,11]. However, despite the recognition of several strong risk factors for suicide such as mental disorders, alcohol and drug use, physical injuries, and intoxication or overdose, several studies indicate that suicidal ideation and planning are not always readily identified among the ED users [12-15]. There are indications that frequent users of ED have increased mortality due to drug intoxication and suicide [6,16]; if confirmed in prospective investigations these systemic factors could be easily employed at EDs as a warning sign for potential suicide risk [11].

The ED at Landspitali, the National University Hospital (LUH), is the only ED serving the larger Reykjavik capital area. Thanks to the universally used personal identification number and population registries, e.g. on causes of death and healthcare utilization, this population-based cohort can be prospectively followed and provides ideal circumstances to study risk factors for suicide, with epidemiological methods. Leveraging these resources, the aim of our study was to evaluate whether the number of visits to the ED is associated with completed suicide, while taking into consideration known risk factors.

## Methods

This is a population-based case–control study nested in the cohort of those attending the ED at LUH. The case–control approach was used to be able to investigate simultaneously many possible risk factors for suicide. The primary source of data was computerized records of attendees discharged home, i.e. not admitted to one of the hospital wards, over an inclusion period 2 April 2002 to 31 December 2008. The records contain routinely collected data on every visit of ED attendees18 years or older, including the unique registration number of each visit, personal identification number according to the National Registry, birth date, gender, admission date, main discharge diagnosis according to ICD-10 (as diagnosed by the attending physician), and discharge date. No obligatory referral system was in operation and the study was confined to new attendances; no visits by appointment are included.

The ED at the LUH is the only general ED and acute care hospital operated for adults in the larger capital area of Reykjavik (the municipalities of Reykjavik, Kopavogur, Seltjarnarnes, Gardabaer, Hafnarfjordur, Alftanes, and Mossfellsbaer) during the study period. The LUH is owned and operated by the government, and is the nation’s main teaching hospital for medicine, nursing and other healthcare professionals. At the LUH there are other EDs for psychiatry, gynaecology and obstetrics; and in addition within the primary health care system to these services there were access to out of hospital specialist service, and to general practitioners. Healthcare services are financed by state taxes, and all residents are covered by the national health insurance schemes that pay the bulk of the patients’ costs. The number of patients attending the ED, the cohort, comprised 107,190 patients making 258,025 visits. In 2005, the mid-year population aged 18 years and older of the Reykjavik capital area was 137,124 [17]. So the people attending the ED during the study period compose 78% of the area’s inhabitants, thus the cohort of attendances may be considered population-based.

### Cases

The study is prospective as the data on exposure and outcome are routinely registered independently of each other, and in real time. Every inhabitant of Iceland receive a personal identification number at birth (or at immigration), and these were used at the ED and in the National Cause-of-Death Registry. Personal identification numbers of those visiting the ED (exposure ascertainment) were used in record linkage to the National Cause-of-Death Register in order to identify suicide. The nationwide National Cause-of-Death Registry records information based on death certificates and vital status was ascertained for all through the registry during the follow-up period 2002 to 2008 [17]. Suicide cases were defined as persons whose cause of death was in the categories: Suicide and intentional injuries (ICD-10 codes X60-X84), or Injuries of undetermined intent (ICD-10 codes Y10-Y34). This procedure ensured that all people who had died from suicide in the cohort during 2002 to 2008 were included as cases, altogether 152 persons. One hundred and ninety people died of the same diagnoses in the capital area of Reykjavik in this period according to the National Cause-of-Death Registry [17], so the 152 cases in the study represent 80% of the suicide cases in the geographically and time-framed population of the catchment area of the ED in the study.

### Controls

The controls were chosen from the unique set of people attending the ED, who were at risk of becoming a case (die by suicide) at the precise time each case died, according to the description by Rothman [18]. This set, which changes from one case to next, is called risk set for the case. For every case we randomly selected 10 controls from the risk sets. The exposure variables, the different discharge diagnoses and number of visits to the ED, were counted up to the day of death of the cases, and the index day of the controls. This procedure ensures that the controls represent the exposure condition of those attending the ED [19].

### Assessment of exposure

Some of the ED users made a number of visits to the department each year or in different years during the inclusion period of the cohort. Only visits to the ED, which ended in discharge, are included in the number of visits. The total number of visits was counted per individual starting with the first visit and ending at the day of death or the corresponding index-day for controls. The number of visits due to Injuries, poisoning, and certain other consequences of external causes (ICD-10, codes S00-T98) were similarly counted. At the time of discharge from the ED the attending physician chose one diagnosis as the main one to be recorded in the computer file and these were used for the diagnostic information in the study. The exposure categories were designed according to these main diagnoses, which, in turn, were according to ICD-10. The main diagnoses as exposure categories were registered as ever/never per individual. At the ED the categories Injury, poisoning and certain other consequences of external causes, ICD-10 codes S00 to T98 were used, but not External causes of injury and poisoning, ICD-10 codes V01 to Y98. Diagnosis or diagnostic categories of Mental and behavioural disorders (ICD-10 codes F00-F99), Symptoms, signs, and abnormal clinical and laboratory findings (ICD-10 codes R00-R99), and Poisoning by drugs, medicaments and biological substances (ICD-10 codes T36-T50) were a priori defined as important exposure categories consistent with previous report [20]. Several other diagnostic categories were used at discharge from the ED and were considered and are shown for completeness.

### Data analysis

A logistic regression analysis was performed, where the case–control status was the dependent variable [21]. Age was treated as a continuous variable expressed in years, and gender as a dichotomous variable. The number of visits to the ED was treated as a continuous variable, the highest number of visits truncated at ten or more. Whether an individual ever or never received the particular main diagnosis or diagnostic category was treated as a dichotomous variable. We did several calculations: comparison between cases and controls without any adjustment, and then adjusted for age and gender to evaluate the different main discharge diagnoses and the number of visits to the ED as a continuous variable. In separate analyses the numbers of visits, and number of visits due to injuries, were evaluated as a categorical variable adjusted for age, gender, mental disorder, non-causative diagnosis, and poisoning. Separate analyses were done after stratifying by gender and in additional separate analyses after dividing the cases into two groups according to whether defined by the death certificates as Suicide and intentional injuries or Injuries of undetermined intent in introducing the number of visits as a continuous variable.

The statistical analyses were performed using the PASW (SPSS) software version 18, and Microsoft Excel 2007.

The National Bioethics Committee (VSNb2009020009/03.7), the Ethical Committee of the Landspitali University Hospital, and the Data Protection Commission (2009020152BRA/-) approved the study.

## Results

The means of self-inflicted injury and events of undetermined intent according to the death certificates among cases are shown in Table 1. The most common means was drug intoxication or 46.1%, 25.7% was hanging, strangulation and suffocation, and 8.6% was by means of firearm.

**Table 1:** **Cause of death among cases according death certificates, Suicide and intentional injuries (ICD-10 codes X60-X84), or Injuries of undetermined intent (ICD-10 codes Y10-Y34)**

**By means of - (ICD-10)**	**Suicide (n = 108)**	**Undetermined intent (n = 44)**
	**N (%)**	**N (%)**
- nonopioid analgesics, antipyretics, and antirheumatics (X60 or Y10)	3 (3)	4 (9)
- antiepileptic, sedative-hypnotic, antiparkinsonism, and psychotropic drugs (X61 or Y11)	21 (19)	19(43)
- narcotics, and psychodysleptics (hallucinogens) (X62 or Y12)	2 (2)	15 (34)
- other and unspecified drug, medicaments, and biological substances (X64 or Y14)	4 (4)	2 (5)
- alcohol (X65 or Y15)	0	1 (2)
- exposure to other gases and vapours (X67 or Y17)	6 (6)	0
- other and unspecified chemicals and noxious substances (X69 or Y19)	0	1 (2)
- hanging, strangulation, and suffocation (X70 or Y20)	39 (36)	0
- drowning, and submersion (X71 or Y21)	6 (6)	0
- rifle, shotgun, and larger firearm discharge (X73 or Y23)	9 (8)	0
- other and unspecified firearm discharge (X74 or Y24)	4 (4)	0
- smoke, fire, and flames (X76 or Y26)	1 (1)	0
- sharp object (X78 or Y28)	3 (3)	0
- jumping from a high place (X80 or Y30)	6 (6)	0
- crashing of motor vehicle (X82 or Y32)	2 (2)	0
- unspecified means (X84 or Y34)	2 (2)	2 (5)

Table 2 shows the baseline characteristics of cases and controls and Table 3 shows selected diagnoses at baseline among cases and controls. These diagnoses or diagnostic categories were counted as ever occurring as a main diagnosis. Injury and poisoning (ICD-10 codes S00-T98) were the most common diagnoses among both cases and controls, followed by non-causative diagnosis (ICD-10 codes R00-R99), and diseases of the musculoskeletal system (ICD-10 codes M00-M99). Diagnoses in the category mental disorders (ICD-10 code F00-F99) were more common among cases than controls, 16% versus 2%, which was also true for non-causative diagnosis (ICD-10 codes R00-R99) 35% versus 14%, and for poisonings (ICD-10 codes T36-T50) 5% versus 0%.

**Table 2:** **Characteristics of cases and controls, and number of visits to the emergency department, univariate odds ratio (OR), 95% confidence intervals, and p-values**

	**Cases (n = 152)**	**Controls (n = 1520)**		
	**N (%)**	**N (%)**	**OR (95% CI)**	**p-value**
Age				
Mean (standard deviation)	42 (15)	43 (18)	1.00 (0.99-1.01)	0.79
Median, IQR (Lower, Higher)	43 (29, 53)	39 (27, 56)		
Gender				
Male	103 (68)	817 (54)	1.00 Referent	
Female	49 (32)	703 (46)	0.55 (0.39-0.79)	0.001
Number of visits				
1	49 (32)	869 (57)	1.00 Referent	
2	21 (14)	335 (22)	1.11 (0.66-1.88)	0.69
3	22 (14)	163 (11)	2.39 (1.41-4.07)	0.001
4	18 (12)	71 (5)	4.50 (2.49-8.13)	<0.001
5	6 (4)	28 (2)	3.80 (1.50-9.61)	0.005
6	7 (5)	20 (1)	6.21 (2.51-15.38)	<0.001
7	7 (5)	12 (1)	10.35 (3.90-27.44)	<0.001
8	6 (4)	3 (0)	35.47 (8.61-146.08)	<0.001
9	3 (2)	3 (0)	17.74 (3.49-90.15)	0.001
10 or more	13 (9)	16 (1)	14.41 (6.56-31.64)	<0.001

**Table 3:** **Number of selected diagnoses among cases and controls at discharge from the emergency department**

**Diagnoses (ICD-10)**	**Cases**	**Controls**
	**(n = 152)**	**(n = 1520)**
	**N (%)**	**N (%)**
Certain infectious and parasitic diseases (A00-B99)	5 (3)	40 (3)
Neoplasms (C00-D48)	0 (0)	4 (0)
Endocrine, nutritional and metabolic diseases (E00-E90)	3 (2)	11 (1)
Mental and behavioural disorders (F00-F99)	24 (16)	36 (2)
Mental and behavioural disorders due to psychoactive substance use (F10-F19)	16 (11)	21 (1)
Mental and behavioural disorders not due to psychoactive substance use (F other than F10-F19)	10 (7)	15 (1)
Diseases of the nervous system (G00-G99)	3 (2)	28 (2)
Diseases of the eye and adnexa (H00-H59)	6 (4)	36 (2)
Diseases of the ear and mastoid process (H60-H95)	6 (4)	15 (1)
Diseases of the circulatory system (I00-I99)	10 (7)	87 (6)
Diseases of the respiratory system (J00-J99)	12 (8)	62 (4)
Diseases of the digestive system (K00-K93)	10 (7)	69 (5)
Diseases of the skin and subcutaneous tissue (L00-L99)	19 (13)	78 (5)
Infections of the skin and subcutaneous tissue (L00-L08)	14 (9)	67 (4)
Diseases of the musculoskeletal system and connective tissue (M00-M99)	38 (25)	213 (14)
Dorsalgia (M54)	10 (7)	44 (3)
Diseases of the genitourinary system (N00-N99)	6 (4)	60 (4)
Symptoms, signs and abnormal clinical and laboratory findings (R00-R99)	53 (35)	262 (17)
Pain in throat and chest (R07)	12 (8)	52 (3)
Abdominal and pelvic pain (R10)	13 (9)	67 (4)
General symptoms and signs (R50-R69)	20 (13)	73 (5)
Injury, poisoning and certain other consequences of external causes (S00-T98)	103 (68)	943 (62)
Injury to certain body regions (S00-S99)	58 (38)	634 (42)
Superficial injury to head (S00)	12 (8)	45 (3)
Open wound of head (S01)	19 (13)	85 (6)
Fracture of skull and facial bones (S02)	4 (3)	8 (0)
Fracture of ribs (S22.3)	10 (7)	38 (3)
Injuries to multiple body regions, burns, poisonings, and toxic effects (T00-T98)	28 (18)	122 (8)
Poisoning by drugs, medicaments and biological substances (T36-T50)	9 (6)	8 (0)
Factors influencing health status and contact with health services (Z00-Z99)	9 (6)	72 (5)
Procedure not carried out because of patient’s decision for other and unspecified reason (Z53.2)	5 (3)	38 (3)
Ever discharged without diagnosis	35 (23)	233 (15)

Table 4 shows the odds ratio (OR) and the 95% confidence intervals (CI) for suicide according to selected main discharge diagnoses adjusted for age and gender, and separately when adjusted for age, gender, and number of visits to the ED. Several diagnoses were strongly associated with suicide risk in the analysis when adjusted for age, and gender; however the ORs were generally lower and mostly statistically non-significant when also adjusted for number of visits. When dividing the category of mental disorders into the subcategories disorders due to psychoactive substance use (ICD-10 codes F10-F19), and disorders not due to psychoactive substance use (ICD-10 codes F other than F10-F19), the ORs were of similar size in both analyses. Three categories of diagnoses were significantly associated with suicide in the analysis when adjusting for number of visits to the ED, i.e. mental disorders (ICD-10 codes F00-F99), non-causative diagnosis (ICD-10 codes R00-R99), and poisoning by drugs (ICD-10 codes T36-T50).

**Table 4:** **Adjusted odds ratio (OR), 95% confidence intervals (CI), and p-values of suicide risk according to ever a discharge diagnosis in each category**

**Diagnosis at discharge (ICD-10)**	OR (95% CI)*	**p-value**	OR (95% CI)**	**p-value**
Certain infectious, and parasitic diseases (A00-B99)	1.28 (0.49 – 3.31)	0.61	0.61 (0.22 – 1.73)	0.36
Endocrine, nutritional, and metabolic diseases (E00-E90)	3.14 (0.85 – 11.54)	0.09	1.60 (0.36 – 6.50)	0.51
Mental, and behavioural disorders (F00-F99)	7.70 (4.43 – 13.38)	<0.001	3.08 (1.61 – 5.88)	0.001
Mental, and behavioural disorders due to psychoactive substance use (F10-F19)	8.02 (4.07 – 15.83)	<0.001	2.78 (1.09 – 6.20)	0.013
Mental and behavioural disorders not due to psychoactive substance use (F other than F10-F19)	7.60 (3.31 – 17.43)	<0.001	3.42 (1.34 – 8.72)	0.01
Diseases of the nervous system (G00-G99)	1.10 (0.33 – 3.69)	0.88	0.54 (0.14 – 2.11)	0.38
Diseases of the eye, and adnexa (H00-H59)	1.81 (0.74 – 4.41)	0.19	1.51 (0.60 – 3.83)	0.39
Diseases of the ear, and mastoid process (H60-H95)	4.13 (1.57 – 10.91)	0.004	2.04 (0.65 – 6.41)	0.22
Diseases of the circulatory system (I00-I99)	1.16 (0.58 – 2.32)	0.68	0.44 (0.20 – 0.98)	0.044
Diseases of the respiratory system (J00-J99)	2.27 (1.19 – 4.36)	0.013	1.27 (0.61 – 2.64)	0.53
Diseases of the digestive system (K00-K93)	1.57 (0.79 – 3.14)	0.20	0.70 (0.32 – 1.51)	0.36
Diseases of the skin, and subcutaneous tissue (L00-L99)	2.55 (1.49 – 4.36)	0.001	1.53 (0.84 – 2.78)	0.17
Infections of the skin, and subcutaneous tissue (L00-L08)	2.09 (1.14 – 3.83)	0.017	1.31 (0.67 – 2.56)	0.43
Diseases of the musculoskeletal system and connective tissue (M00-M99)	2.06 (1.39 – 3.07)	<0.001	1.23 (0.79 – 1.92)	0.36
Dorsalgia (M54)	2.50 (1.23 – 5.12)	0.012	1.62 (0.74 – 3.53)	0.23
Diseases of the genitourinary system (N00-N99)	1.07 (0.45 – 2.53)	0.88	0.89 (0.37 – 2.15)	0.79
Symptoms, signs, and abnormal clinical and laboratory findings (R00-99)	2.87 (1.99 – 4.15)	<0.001	1.60 (1.06 – 2.43)	0.027
Pain in throat, and chest (R07)	2.43 (1.26 – 4.70)	0.008	1.00 (0.46 – 2.17)	1.0
Abdominal, and pelvic pain (R10)	2.46 (1.30 – 4.64)	0.005	1.37 (0.69 – 2.74)	0.37
General symptoms, and signs (R50-R69)	3.23 (1.89 – 5.52)	<0.001	1.60 (0.88 – 2.93)	0.12
Injury, poisoning, and certain other consequences of external causes (S00-T98)	1.24 (0.86 – 1.79)	0.24	0.84 (0.56 – 1.24)	0.37
Injury to certain body regions (S00-S99)	1.12 (0.78 – 1.59)	0.55	0.75 (0.52 – 1.10)	0.14
Superficial injury to head (S00)	2.72 (1.40 – 5.29)	0.003	1.71 (0.82 – 3.56)	0.16
Open wound of head (S01)	2.34 (1.38 – 3.98)	0.002	1.43 (0.80 – 2.58)	0.23
Fracture of skull and facial bones (S02)	4.35 (1.28 – 14.83)	0.019	2.80 (0.68 – 11.59)	0.16
Fracture of ribs (S22.3)	2.43 (1.18 – 5.00)	0.016	1.61 (0.73 – 3.55)	0.24
Injuries to multiple body regions, burns, poisonings, and toxic effects (T00-T98)	2.52 (1.60 – 3.97)	<0.001	1.41 (0.85 – 2.33)	0.18
Poisoning by drugs, medicaments and biological substances (T36-T50)	12.89 (4.81 – 34.52)	<0.001	5.08 (1.69 – 15.25)	0.004
Factors influencing health status and contact with health services (Z00-Z99)	1.27 (0.62 – 2.60)	0.52	0.57 (0.26 – 1.25)	0.16
Procedure not carried out because of patient’s decision for other and unspecified reason (Z53.2)	1.33 (0.51-3.45)	0.56	0.59 (0.19-1.60)	0.28
Ever discharged without diagnosis	1.60 (1.07 – 2.40)	0.022	0.73 (0.45 – 1.18)	0.20

*Adjusted for age and gender, **Adjusted for age, gender and number of visits.

Table 5 shows the ORs and 95% CI for suicide according to number of all visits to the ED with three levels of adjustments, for age, and gender, for age, gender, and mental disorders, and for age, gender, mental disorders, and non-causative diagnosis, and poisoning. In these three analyses there was an increase of suicide risk with increasing number of visits. The OR for four visits to the ED was 2.98 (95% CI 1.57-5.65), and for seven visits the OR was 5.97 (95% CI 2.11-16.93).

**Table 5:** **Adjusted odds ratio (OR), 95% confidence intervals (CI), and p-values of suicide risk according to number of visits to the emergency department**

**Number of visits**	OR (95% CI)*	**p-value**	OR (95% CI)**	**p-value**	OR (95% CI)***	**p-value**
1	1.00 Referent		1.00 Referent		1.00 Referent	
2	1.12 (0.66 – 1.91)	0.66	1.06 (0.62 – 1.80)	0.84	0.96 (0.56 – 1.65)	0.89
3	2.40 (1.41 – 4.09)	0.001	2.37 (1.39 – 4.31)	0.002	2.17 (1.26 – 3.73)	0.005
4	4.38 (2.42 – 7.95)	<0.001	3.73 (2.02 – 6.89)	<0.001	2.98 (1.57 – 5.65)	0.001
5	3.74 (1.47 – 9.50)	0.006	3.29 (1.28 – 8.49)	0.014	2.60 (0.98 – 6.91)	0.055
6	6.38 (2.56 – 15.93)	<0,001	4.96 (1.92 – 12.83)	0.001	3.26 (1.15 – 9.20)	0.026
7	10.26 (3.83 – 27.50)	<0.001	8.53 (3.12 – 23.30)	<0.001	5.97 (2.11 – 16.93)	0.001
8	35.35 (8.45 – 147.87)	<0.001	25.44 (5.83 – 111.03)	<0.001	17.78 (4.01 – 78.83)	<0.001
9	20.69 (4.01 – 106.79)	<0.001	17.26 (3.26 – 91.37)	0.001	12.18 (2.14 – 69.44)	0.005
10 or more	13.59 (6.15 – 30.04)	<0.001	8.34 (3.48 – 19.99)	<0.001	5.20 (2.04 – 13.23)	0.001

*Adjusted for age, and gender.

**Adjusted for age, gender, and mental and behavioural disorders as the main discharge diagnosis.

***Adjusted for age, gender, mental and behavioural disorders, symptoms, signs, and abnormal clinical and laboratory findings, and poisoning by drugs, medicaments and biological substances as the main discharge diagnoses.

The ORs and 95% CI for suicide according to number of visits due to injury, poisoning, and certain other consequences of external causes (S00-T98) to the ED were estimated separately with three levels of adjustments, for age, and gender, for age, gender, and mental disorders, and for age, gender, mental disorders, and non-causative diagnosis, and poisoning. In these three analyses there was an increase of suicide risk with increasing number of visits.

When stratifying by gender and then introducing the number of visits to the ED as a categorical variable, the OR increased with increasing number of visits in a similar way for both genders as in the total material adjusted for age, mental and behavioural disorders, non-causative diagnosis, and poisoning. The 95% CIs following these ORs were wider, and the ORs for women were somewhat higher than for men.

Similar results were obtained when dividing the cases into two groups, i.e. definite Suicide and intentional injuries (ICD-10 codes X60-X84) or Injuries of undetermined intent (ICD-10 codes Y11-Y34), compared to the results obtained when analysing these as one outcome. The OR was 1.28 (95% CI 1.16-1.41) for Suicide and intentional injuries, and 1.31 (95% CI 1.15-1.48) for Injuries of undetermined intent compared with controls, adjusted for age, gender, number of visits (as continuous variable), mental and behavioural disorders, non-causative diagnosis, and poisoning as the main discharge diagnosis. For all cases combined the OR in similar analysis with adjustment for same variables was 1.29 (95% CI 1.19-1.40).

## Discussion

Our population-based study shows that the number of visits to the ED is an independent risk factor for suicide when several other well-known risk factors for suicide are taken into account, including attendances due to psychiatric diagnoses. The risk for suicide increased with increasing number of visits to the ED in a dose–response manner. The number of visits to the ED can thus be seen as a risk indicator on a complex path to a completed suicide [22]. Thus, the suicide risk related to the number of visits to the ED may open new intervention measures for suicide prevention. The relationship of the suicide to the increasing number of visits was not uniform, but a presence of threshold was observed at two visits, which coincided with the mean number of visits among the controls. The suicide risk increased nearly consistently for more than two numbers of visits up to eight visits. Few visits during the study period were common among cases; 40% of cases attended the ED four times or more compared to 10% among controls. The results confirm indications from a prior register study from Alberta, Canada, where the number of visits to ED seemed to be associated with suicide [6].

The number of male cases was double the number of female cases; however, this difference was adjusted for in the design of the study (numerous controls of both genders), and in the multivariate analyses, and when stratifying by gender, similar patterns of suicide risk were obtained concerning the most important risk factors studied. When taking into consideration that the identification of the cases was based on two categories of causes of death, namely, Suicide and intentional injuries, and Injuries of undetermined intent, the calculation yielded similar OR as found when analysing all cases together. Thus, it seems justified to combine these diagnoses in the present study on risk factors for suicide.

The patients in the present study are confined to those discharged home from the ED after clinical work up and initial treatment. Visits of patients who were admitted to a hospital ward when attending the ED were not included in this study and these differ from those who are discharged home. Thus one may postulate that when patients were admitted, they were considered seriously ill or with severe injuries, and they were under observation and treatment for days, while those treated only at the ED did not stay there for more than some hours. The physicians of the ED are responsible for the diagnosis and treatment of the discharged patients; however, in case of self-harm or suspicion of suicidality, a psychiatrist is routinely consulted before eventual discharge.

The universal use of the personal identification number in the files of the ED enabled an accurate registration of whether and when the patients made repeated visits to the ED through record linkage, thus counting the number of visits and identifying the discharge diagnosis. The personal identification number of the patients at the ED was also used in record linkage with the National Cause-of-Death Registry to identify suicide cases. The setting is favourable for counting the number of visits to the ED and the discharge diagnosis, as the ED and the hospital were the only acute healthcare institutes of this kind serving the population in the catchment area, so they did not have any competition from other similar institutions.

### Limitation

The study material originates from a single academic health care institution in a capital area serving as the number one trauma centre and a community hospital. This may limit the generalizability of the results, but the size and the characteristics of the background population are known and the population is homogenous, being 99% white Caucasian, and there was a uniform financing of healthcare and insurance.

In the study, the cumulative information from the ED and the comprehensive population registries is used in a prospective design. The definition of the cases is based on information from death certificates, which are issued by physicians. Information on the quality of the recording of the cause of death on death certificates in Iceland is not at hand; however, an evaluation of registration data from many countries has classified the data from Iceland as high-quality overall, and it was ranked in the same category as data from several developed countries including the USA and the UK [23]. There have been claims of underreporting of suicide in official data, and that may be an inherent weakness of studies relying on such information. Such misclassification, if present in the study, would bias the observed associations toward the null value if non-differential with respect to number of ED visits. Knowledge on how frequently a patient has visited the ED prior to death may be unimportant for the physician, who is attesting the death certificate, as the association of number of visits and suicide risk has only been recently discussed [6]. Serious bias due to these circumstances is unlikely given the high ORs observed for multiple ED visits.

The possible time-dependent relation between attendances and suicide risk was not investigated in the present study, and calls for larger material and different design.

As ICD-10 codes of external causes of injury and poisoning were not used when registering the main diagnoses at the ED, it was not possible to take diagnosed self-harm into consideration. Another limitation of this study is the sole use of the main diagnosis at discharge from the ED, and that we have only taken it into account as an ever/never phenomenon. Many of the users of the ED surely also had other diagnoses in the paper records not registered in the computerized records. Nevertheless, the main diagnosis at discharge is considered to reflect the main clinical evaluation of the attending physician taking into account the patient’s complaints and condition on the visit.

## Conclusions

Number of visits to the ED is an independent risk factor for suicide adjusted for other known and important risk factors. The prevalence of four or more visits was 40% among cases compared with 10% among controls. This new risk factor may open new venues for suicide prevention.

The clinical implication is that patients with many visits to the ED, particularly four or more visits during a period of a few years, should be evaluated in more detail with respect to potential suicide ideation or intention. This new marker for suicide risk does not replace previously known risk factors for suicide but can be viewed as a new supplementary factor, facilitating identification of potential suicide victims at the ED. Further studies are needed into the practical usefulness of of this finding.
